# The impact of diurnal variation on induced sputum cell counts in healthy adults

**DOI:** 10.1186/2045-7022-3-8

**Published:** 2013-03-28

**Authors:** Warren J Davidson, Lisa E Wong, Stephanie The, Richard Leigh

**Affiliations:** 1 Department of Medicine, University of Calgary, 7007-14th Street SW, Calgary, Alberta, T2V 1P9, Canada; 2 Faculty of Kinesiology, University of Calgary, Calgary, Alberta, Canada

**Keywords:** Induced sputum, Inflammation, Diurnal variation

## Abstract

**Background:**

Induced sputum cell counts are a non-invasive, reliable method for evaluating the presence, type, and degree of inflammation in the airways of the lungs. Current reference values for induced sputum cell counts in healthy adults do not account for the effects of circadian rhythm, including diurnal variation. The objective of this study was to describe the diurnal variation in induced sputum cell counts, compared between early morning and late afternoon, in healthy adult individuals.

**Methods:**

100 healthy adult subjects with no history of lung disease and normal bronchial reactivity proceeded with induced sputum testing at 7 am and 4 pm on different days. The order of testing was randomized. The cytotechnologist preparing and performing the cell counts was blinded to the sample collection time and subject characteristics.

**Results:**

65 subjects were included in the final analyses. There was no significant change in the total and differential sputum cell counts between the 7 am and 4 pm collections. There was good inter-observer agreement with respect to differential sputum cell count interpretation.

**Conclusions:**

This is the largest study to assess the variation in induced sputum cell counts in healthy adult subjects at different times of the day. We found no significant change in total and differential sputum cell counts between the 7 am and 4 pm collection time points. This is in contrast to studies in asthmatics that demonstrated a circadian variation in sputum cell counts and other markers of inflammation, suggesting that fluctuations in airway inflammatory cells during the day are a disease-specific effect.

## Background

Induced sputum cell counts are a non-invasive, reliable method for evaluating the presence, type, and degree of inflammation in the airways of the lungs [1-3]. Sputum cell counts can be used clinically to assist with management of underlying airway inflammation, including titration of inhaled corticosteroid therapy in patients with asthma [4]. Tailoring asthma management based on sputum eosinophil counts has been shown to be effective in reducing asthma exacerbations [4-7] and is now considered standard of care for adults with moderate to severe asthma in the most recent iteration of the Canadian asthma guidelines [8].

As in many other biological systems, circadian rhythms are known to have an impact on the respiratory system. In healthy subjects, endogenous circadian rhythms contribute to diurnal changes in pulmonary function [9]. In asthmatic patients, there is a circadian influence on airway resistance and lung volumes [10,11]. Moreover, asthmatics have been shown to have decreased lung function at night and early morning [12]. Analysis of bronchoalveolar lavage fluid in patients with asthma has revealed a circadian alteration in lymphocytes and a variety of cytokines [13,14]. One small study examining the circadian changes in induced sputum in 11 asthmatic patients demonstrated an increase in early morning total sputum leukocytes and eosinophils [15]. Whether these changes are a normal response or specific to asthma remains to be determined. Current reference values for induced sputum cell counts in healthy adults do not account for the effects of circadian rhythm [16,17]. Thus, it is necessary to understand the effect of normal circadian variation, including diurnal variation, on induced sputum cell counts in healthy non-asthmatic individuals, especially when testing for clinical or research purposes can be performed at various times throughout the day. Understanding whether these alterations are disease-specific or simply reflect circadian influences on normal airway biology will improve our interpretation of induced sputum cell counts in the investigation and management of inflammatory airways disorders, and will further standardize clinical and research methodologies. Therefore, the aim of this study was to evaluate the diurnal changes in induced sputum cell counts in healthy adult subjects. We hypothesized that, similar to patients with asthma, total sputum leukocytes would be elevated in the early morning compared with the evening.

## Methods

### Study subjects

The study was approved by the Institutional Research Ethics Board and written informed consent was obtained from all subjects. Adult study participants were eligible for inclusion into the study, provided they had no history of any chronic respiratory conditions and had normal spirometry (forced expiratory volume in one second [FEV_1_] >80% predicted and forced expiratory volume in one second to forced vital capacity ratio [FEV_1_/FVC] >0.75), with no significant bronchodilator response (FEV_1_ and FVC <200 mL increase and <12% increase following short-acting bronchodilator administration). Potential study participants were excluded from the study if they had any of the following: a positive methacholine challenge test (defined here as a provocative concentration of methacholine required to give a 20% reduction in baseline FEV_1_ (PC_20_) <16 mg/mL); a smoking history ≥5 pack years or had smoked within the past 12 months; a history of atopy; a history of rhinitis symptoms; a diagnosed upper respiratory tract infection 4 weeks prior to study participation; or a history of any inflammatory condition considered by the investigators to be clinically relevant, including any underlying autoimmune disorder, infectious disease, immunodeficiency, malignancy or clinically relevant cardiovascular, neurological, endocrine or hematological disorder. Women who were pregnant, or who were planning on become pregnant during the study period, and women who were breastfeeding were also excluded. Finally, only those study participants who provided written informed consent were included in the study.

### Study design

The primary objective was to describe the diurnal variation in induced sputum cell counts, compared between early morning and late afternoon, in healthy adult individuals.

Study participants attended our laboratory on three separate occasions. During the first visit, the subject’s medical history was reviewed to determine if they met inclusion/exclusion criteria. Pre- and post-bronchodilator spirometry was completed and a methacholine challenge test was performed. Suitable candidates were randomized to undergo initial induced sputum collection at either 7 am or 4 pm. At the second visit subjects performed the first induced sputum test. Subjects were asked to return 2–5 days later to complete the second induced sputum collection. To assess the subjects’ sleep patterns prior to each sputum collection, including any disruption to their usual duration of sleep, the subjects answered standardized questions about their usual weekly sleep habits and their sleep schedule during the study period.

The randomization sequence was computer-generated and stored in opaque, sealed envelopes that were held by an individual not involved in the study.

### Procedures

#### Pulmonary function testing

Spirometry, pre- and post-bronchodilator administration, and methacholine challenge testing were performed according to American Thoracic Society (ATS) guidelines [18,19].

#### Sputum induction and processing

Induced sputum collection was performed using accepted techniques as per standardized guidelines [20]. Sputum was induced with escalating concentrations of hypertonic saline (4%, 4%, 5%). Subjects inhaled the hypertonic saline for 7 minutes for each concentration. If the baseline FEV_1_ fell by >20%, or if the subject experienced respiratory distress, the procedure was discontinued and treatment with inhaled bronchodilator was given. Spirometry was repeated to ensure the FEV_1_ improved to within 10% of baseline. After induction, 10 mL of 10% buffered formalin (Starplex Scientific Inc, Etobicoke, Ont) was added to the sample container, as described previously [21]. Sputum samples were batched in groups of 4 or 6 and sent for processing by a cytotechnologist blinded to the collection times. The expectorated preserved sputum was poured into a petri dish and examined. Colour was noted and dense areas were chosen for processing. The sample was then suspended in phosphate-buffered saline (PBS) and rocked for 10 minutes before being centrifuged (500 rpm × g for 10 min). After centrifugation, the supernatant was removed and the sample was briefly vortexed. These steps were repeated three times. A solution of 2.5% trypsin (3 times volume to sputum weight) was added to the sample and the solution was then incubated at 37°C overnight (16–17 hours). Total cell counts were obtained manually using a Bright-line neubuer hemacytometer (Hausser Scientific, Horsham, PA). The cells were diluted in PBS to 1 × 10^6^/ml and cytospin slides were prepared, before being stained with Congo red, after which differential sputum cell counts were performed on 400 non-squamous cells. Samples were considered adequate for analysis if there was <20% squamous cell contamination. Measurements were compared with standardized values [16]. Cell counts were performed by an experienced cytotechnologist blinded to subject characteristics and sputum sample collection times. To assess inter-observer agreement in the differential sputum cell count, both slides from 20 subjects were recounted by a second experienced cytotechnologist, who was also blinded to subject characteristics and sputum sample collection times. Given that the samples were preserved in formalin, analysis of sputum fluid-phase mediators, including cytokines and chemokines, could not be performed. Cell viability could not be determined.

### Sample size and statistical analysis

Belda et al. measured induced sputum cell counts in healthy adults, without reference to time of sample collection, and found a mean total cell count of 4.13 × 10^6^ cells/gram (standard deviation (SD) 4.8 × 10^6^ cells/gram) [16]. Panzer et al. found an increase in total sputum leukocytes and eosinophils at 7 am compared with 4 pm in patients with mild asthma [15]. Based on the cell counts from the Belda study and the increase in early morning sputum cell counts in the Panzer study, we calculated that 63 subjects would be required to detect a 50% increase in total cell counts in the 7 am collection compared with the 4 pm collection, using a one-sided test, a power of 0.80, and an alpha level of 0.05.

Values from the different sputum collections were compared using the paired t-test for normally distributed data and the Wilcoxon signed rank test for non-parametric data. To assess inter-observer agreement, Bland-Altman plots were performed and the intra-class correlation coefficient was calculated. For the primary outcome of change in total sputum cell count, a p-value <0.05 was considered statistically significant. To account for multiple analyses, a p-value <0.01 was considered statistically significant for the differential sputum cell counts.

## Results

### Study subjects

**Figure 1:**
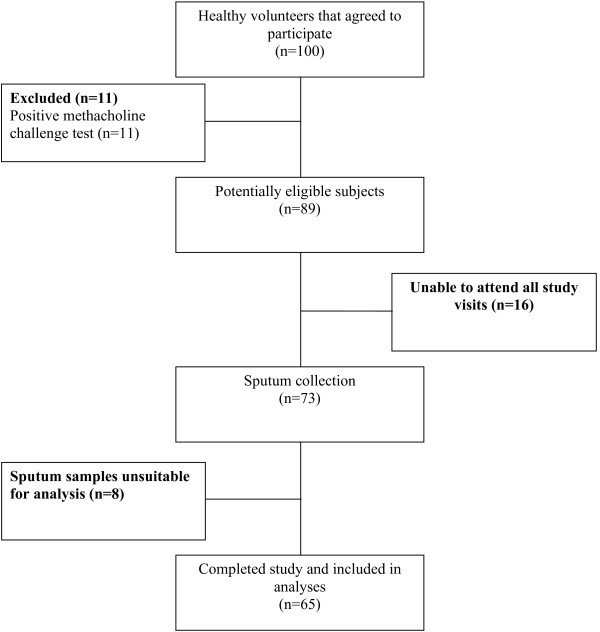
Subject flow.

Subject enrolment started in January 2010 and ended in March 2012. Subject flow is summarized in Figure 1. Of the 100 subjects who volunteered to participate, 11 were excluded due to a positive methacholine challenge test, 16 were withdrawn due to difficulties attending all required study visits, and 8 were excluded as at least one of their sputum samples was unsatisfactory for interpretation. Sixty-five subjects were included in the final analyses. Subject characteristics at baseline are listed in Table 1. The majority of the subjects were Caucasian (n = 50) and female (n = 40). The study population had normal spirometry. None of the subjects were taking regular medications at the time of enrolment.

**Table 1:** Baseline characteristics of subjects

**Characteristic**	**Value**
Age, yr	35(9.8)
Male:Female	25:40
Ethnicity, n (%)	
Caucasian	50 (77)
Asian	10 (15)
Other	5 (8)
BMI, kg/m^2^	29.7 (4.8)
Smoking, Pack-years	0.3 (1)
FEV_1_, L	3.52 (3.13,4.17)
FEV_1_ (% pred)	106 (97,114)
FVC, L	4.42 (3.89,5.52)
FVC (% pred)	109 (98,120)
FEV_1_/FVC, %	0.8 (0.77,0.85)

*Definition of abbreviations*: *BMI* = body mass index, *FEV*_*1*_ = forced expiratory volume in one second, *FRC* = functional residual capacity, *FVC* = forced vital capacity. Parametric data are shown as mean (SD). Non-parametric variables are reported as median (IQR).

### Changes in airway inflammatory cells

Table 2 summarizes the induced sputum cell count results. There was no significant change in the total and differential sputum cell counts between the 7 am and 4 pm collections. Although there appeared to be a change in absolute macrophage cell count, the p-value did not reach our *a priori* cut-off for differential cell counts (p < 0.01) and therefore was considered not to be statistically significant. The time between sputum collections was approximately 5 days [mean 125.4 hours (SD 82.2 hours)].

**Table 2:** Change in sputum cell counts

	**7 am**	**4 pm**	**p-value**
Total cell count, x10^6^/g	2 (0.95,3.55)	1.39 (0.75,2.97)	0.34
Neutrophils, %	51.5 (35,68)	51 (35,68)	0.84
Neutrophils, x10^6^/g	0.81 (0.33,1.37)	0.81 (0.28,1.32)	0.96
Eosinophils, %	0.7(0,2)	0.5 (0,2)	0.36
Eosinophils, x10^6^/g	0.0069 (0,0.049)	0.005 (0,0.027)	0.08
Macrophages, %	39.3 (25,57)	37.3 (20,61)	0.44
Macrophages, x10^6^/g	0.67 (0.30,1.39)	0.44 (0.29,0.73)	0.033
Lymphocytes, %	1 (0,2.5)	0.75 (0,3)	0.46
Lymphocytes, x10^6^/g	0.005 (0,0.51)	0.0076 (0,0.0385)	0.076

Variables reported as median (IQR); p-value derived from the Wilcoxon signed-rank test.

### Inter-observer agreement

**Figure 2:**
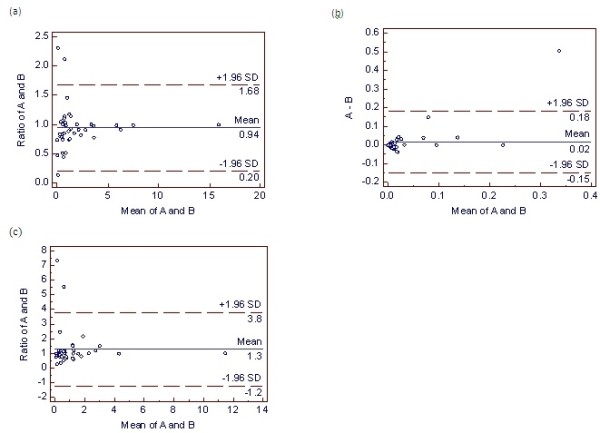
**Bland-Altman plots of the inter-observer agreement for differential sputum cell counts.** Sputum neutrophil, eosinophil, and macrophage cell counts are represented in plots (**a**), (**b**), and (**c**), respectively. The dashed lines represent the inter-observer bias (± 1.96 SD). For the neutrophil and macrophage analyses, the ratio of the measurements was plotted, avoiding the need for log transformation of the data. The eosinophil analysis plotted the difference of the measurements. *Definition of abbreviations*: A = first cytotechnologist cell count; B = second cytotechnologist cell count.

Sputum samples from 20 subjects were randomly selected for differential sputum cell count interpretation by a second cytotechnician blinded to subject characteristics and sputum sample collection times. Bland-Altman analysis revealed good inter-observer agreement (Figure 2 and Table 3). For the neutrophil and macrophage analyses, the ratio of the measurements was plotted, avoiding the need for log transformation of the data. The eosinophil analysis plotted the difference of the measurements. The intra-class correlation coefficient (ICC) revealed very good agreement for the sputum neutrophils and macrophages while there was moderate agreement for the sputum eosinophil counts (Table 3).

**Table 3:** Bland-Altman analysis of inter-observer bias in sputum cell counts

	**Neutrophils (x10**^**6**^**/g)**	**Eosinophils(x10**^**6**^**/g)**	**Macrophages (x10**^**6**^**/g)**
Inter-observer bias (± 1.96 STD)*	0.94 + 0.74	−0.02 ± 0.17	1.3 ± 2.5
ICC (95% CI)	0.998 (0.996 to 0.999)	0.593 (0.231 to 0.785)	0.988 (0.978 to 0.994)

* Ratio of the measurements was plotted for neutrophils and macrophages; difference of the measurements was plotted for eosinophils. *Definition of abbreviations*: *CI* = confidence interval; *ICC* = Intra-class correlation coefficient; *STD* = standard deviation of the difference.

### Sleep questionnaire

Subjects slept for a shorter duration [median 7 hours (IQR 6.1,7.5)] prior to the 7 am collection than the 4 pm collection [median 7.4 hours (IQR 7,8)] (p = 0.0027). During an average week, subjects slept for a shorter duration [7.5 hours (IQR 7,8)] compared with the weekend [median 8 hours (IQR 8,9)] (p < 0.0001). Prior to the 7 am sputum collection, subjects slept for a slightly shorter duration compared with their usual weekday (p = 0.002), while there was no difference in the amount of weekday sleep prior to the 4 pm sputum collection (p = 0.66). Six subjects reported napping during an average week [mean 0.42 hours (SD 0.31 hours)] and 8 subjects during an average weekend [mean 0.91 hours (SD 0.36 hours)]. Ten subjects reported that they perform shift work with 7 subjects reporting working a nightshift prior to one of the sputum collections. However, in this group of subjects there was no difference in the total evening sleep duration before each sputum collection (p = 0.63). No subject napped during the day before the 4 pm collection.

## Discussion

This is the largest study to assess the potential impact of diurnal variation on induced sputum cell counts in healthy subjects at different times of the day. We found no significant change in total and differential sputum cell counts between the 7 am and 4 pm collection times.

Our results differ from the only other study assessing changes in sputum cell counts in healthy controls [22]. Popov and colleagues performed sputum induction at 8 am and 8 pm in 13 asthmatics admitted to hospital for asthma exacerbations and 10 hospital volunteers [22]. In the “control group”, the authors reported a higher total cell count in the morning (median 0.75 × 10^3^ cells/mL, IQR 0.31-2.25 × 10^3^ cells/mL) compared with the evening collection (median 0.33 × 10^3^ cells/mL, IQR 0.1-0.97 × 10^3^ cells/mL, p < 0.001). Sputum eosinophil levels were lower in the evening, whereas macrophages were increased. However, this study had a number of potential limitations, making interpretation of the results difficult. The sample size was small at 10 healthy controls with no justification of the sample size. Inclusion/exclusion criteria for the control subjects were not provided. The collection of the sputum at the different time points did not appear to be randomized and the timing between sputum induction was not described. It was not clear if the cytotechnologist preparing and counting the sputum samples was blinded to the study subjects and collection times. There was no testing for inter-observer agreement. Furthermore, there was no adjustment for multiple comparisons. Our current study now extends our knowledge in this area by clearly controlling for all of these potential limitations present in this earlier study.

Previous studies have shown diurnal variation in pulmonary function in healthy subjects [23,24]. Spengler et al. found that endogenous circadian rhythms, rather than diurnal alterations in behavior or environment, are factors in these diurnal changes [9]. There have been many studies examining the effect of circadian variation in asthma-related inflammation, including induced sputum [15], bronchoalveolar lavage fluid inflammatory cells and cytokines [13,25], systemic inflammatory markers [26,27], and transbronchial biopsies [28]. Our finding of an absence of any significant change in sputum cell counts in healthy subjects at different time points during the day suggests that the circadian/diurnal variation in inflammation previously reported in asthmatic patients is intrinsic to the disease.

In an effort to reduce systematic bias, we randomized the start time of the first induced sputum collection. Although the respiratory therapist performing the induced sputum test could not be blinded, the cytotechnologist preparing the samples and performing the cell counts was blinded to collection time. Use of the formalin preservation technique allowed us to keep the cytotechnician blinded. That is, without the formalin preservation, the sample would have been processed within 2 hours of collection, alerting the cytotechnician to the collection time. The good inter-observer agreement confirmed that the differential sputum cell count results were reliable. We scheduled the second induced sputum collection more than 48 hours after the first collection as repeated induced sputum procedures within 48 hours have been shown to alter the cellular composition of sputum [29-31]. To minimize the chance of external factors altering the sputum composition between collections, we requested that the second collection occur within 5 days of the first test. From the sleep data collected, we did not find a significant difference in the amount of sleep prior to each of the collections. Furthermore, no subjects napped during the day prior to the 4 pm collection. Subjects did sleep approximately 30 minutes less than a usual weekday prior to the 7 am test but this was not surprising, as they were required to be in the laboratory just prior to 7 am.

Given the need for a delayed sputum analysis, we gently mixed all sputum samples with 10% buffered formalin immediately after collection. The use of formalin as a preservation method for induced sputum processing has been validated previously [32]. Specifically, Kelly et al. compared two methods of sputum processing: (1) a (para)formaldehyde-dithiothreitol (DTT) mixture dispersed with trypsin (preservation method), and (2) immediate processing with DTT (routine method). Good agreement between the techniques was found, along with good within-method repeatability for the preservation method, thereby confirming that this method is valid and reliable. Further, no increase in degenerate changes and no interference of cell counts by debris were found [33]. This technique has subsequently been successfully used to process sputum samples in a remote collection site [34]. Additionally, this method of preservation has been shown to have no adverse effect on sample quality and sputum cell counts [21]. Viability of the preserved cells could not be determined. The standard method of measuring cell viability using the trypan blue exclusion technique [35] was not possible as the formalin resulted in cell death. Formalin preserved cells have good morphology and cell counts, total and differential, may be performed in the same way as for the DTT method [32].

Current reference values for absolute and percentage induced sputum cell counts in healthy adults were derived from two major studies [16,17]. In our study, the sputum absolute and percentage cell counts most closely resembled those of Belda and colleagues [16]. We found a slightly lower absolute and percentage neutrophil count. Eosinophil cell count, absolute and percentage, were only slightly higher in our study, as was the variability in the range of these values. Compared with the findings of Spanevello et al. [17], our results showed a slightly higher percentage of sputum neutrophils and a lower percentage of macrophages. The differences may be a result of the formalin fixation method used in our study. While DTT may reduce cell viability [36,37], formalin rapidly inactivates cellular enzymes and may stabilize the cell membrane when added to induced sputum samples [32]. It is possible that use of the preservation technique may have improved cell viability and cellular recovery compared with previous studies reporting normal cell count values.

There are several study limitations. First, the time points chosen were limited by availability of the laboratory to perform induced sputum collection during routine working hours. We cannot comment whether the cellular composition of sputum changes at other points during the very early morning or late evening. However, on a practical basis, it is likely that sputum induction and collection is most commonly performed during daytime hours. Second, given that we required delay sample analysis, we had to preserve the samples. As a result, we were not able to assess whether sputum fluid-phase mediators, such as sputum cytokines, normally vary during the day. Third, although subjects were excluded if they had a history of atopy, we did not perform allergy skin prick testing on all study subjects. Thus, it is possible that some patients may have had underlying atopy with no clinical history.

## Conclusion

This is the largest study to assess the variation in induced sputum cell counts in healthy adult subjects at different times of the day. We found no significant change in total and differential sputum cell counts between the 7 am and 4 pm collection time points. This is in contrast to studies in asthmatics that have demonstrated a circadian variation in sputum cell counts and other markers of inflammation, suggesting that fluctuations in airway inflammatory cells during the day are a disease-specific effect. Whether other markers of airway inflammation exhibit circadian variation in healthy subjects remains to be determined. Our results provide evidence that the timing of daytime induced sputum collection, by itself, does not affect sputum cell count results.

### Ethics approval

Institutional Research Ethics Board, University of Calgary.

Approval reference number: E-22460.

## Abbreviations

ATS: American thoracic society; DTT: Dithiothreitol; ICC: Intra-class correlation coefficient; IQR: Interquartile range; FEV1: Forced expiratory volume in one second; FVC: Forced vital capacity; PBS: Phosphate-buffered saline; PC20: Provocative concentration of methacholine required to give a 20% reduction in baseline FEV_1_; SD: Standard deviation.

## Competing interests

The authors have no potential competing interest related to this study.

## Authors’ contributions

WJD participated in study design, subject recruitment, sample collection and analysis, data collection and analysis, and manuscript preparation. LEW participated in subject recruitment, sample collection and analysis, data collection and analysis, and manuscript preparation. ST sample collection and analysis, and manuscript preparation. RL participated in study design, subject recruitment, sample collection and analysis, and manuscript preparation. All authors read and approved the final manuscript.

## Funding

This study was supported by a University of Calgary, Department of Medicine Research Fund Award.
